# Neonatal Metabolic Acidosis in the Neonatal Intensive Care Unit: What Are the Genetic Causes?

**DOI:** 10.3389/fped.2021.727301

**Published:** 2021-10-18

**Authors:** Haiyan Ma, Zezhong Tang, Feifan Xiao, Long Li, Yangfang Li, Wenyan Tang, Liping Chen, Wenqing Kang, Yulan Lu, Xinran Dong, Guoqiang Cheng, Laishuan Wang, Wei Lu, Lin Yang, Qi Ni, Xiaomin Peng, Yao Wang, Yun Cao, Bingbing Wu, Wenhao Zhou, Deyi Zhuang, Guang Lin, Huijun Wang

**Affiliations:** ^1^Department of Neonatology, Zhuhai Women and Children's Hospital, Zhuhai, China; ^2^Department of Pediatrics, Peking University First Hospital, Beijing, China; ^3^Center for Molecular Medicine, Children's Hospital of Fudan University, Shanghai, China; ^4^Department of Neonatology, People's Hospital of Xinjiang Uygur Autonomous Region, Urumqi, China; ^5^Department of Neonatology, Kunming Children's Hospital, Kunming, China; ^6^Department of Neonatology, Jiangxi Maternal Hospital, Nanchang, China; ^7^Department of Neonatology, Jiangxi Provincial Children's Hospital, Nanchang, China; ^8^Department of Neonatology, Children's Hospital Affiliated to Zhengzhou University, Zhengzhou, China; ^9^Department of Neonatology, Children's Hospital of Fudan University, Key Laboratory of Neonatal Diseases, Ministry of Health, Shanghai, China; ^10^Department of Endocrinology and Inherited Metabolic Diseases, Children's Hospital of Fudan University, Shanghai, China; ^11^Xiamen Key Laboratory of Neonatal Diseases, Xiamen Children's Hospital, Xiamen, China

**Keywords:** neonatal metabolic acidosis, neonatal intensive care units, next-generation sequencing, gene, neonate

## Abstract

Neonatal metabolic acidosis (NMA) is a common problem, particularly in critically ill patients in neonatal intensive care units (NICUs). Complex etiologies and atypical clinical signs make diagnosis difficult; thus, it is crucial to investigate the underlying causes of NMA rapidly and provide disorder-specific therapies. Our study aims to provide an overview of the genetic causes of NMA in patients from NICUs. We performed next-generation sequencing (NGS) on neonates with NMA from January 2016 to December 2019. Clinical features, genetic diagnoses, and their effects on clinical interventions were collected for analysis. In the 354 enrolled patients, 131 (37%) received genetic diagnoses; 95 (72.5%) of them were autosomal recessively inherited diseases. Two hundred and fifteen variants spanning 57 genes were classified as pathogenic (P) or likely pathogenic (LP) in 131 patients. The leading cause was metabolic disorders due to 35 genes found in 89 patients (68%). The other 42 NMA patients (32%) with 22 genes had malformations and renal, neuromuscular, and immune-hematological disorders. Seven genes (*MMUT, MMACHC, CHD7, NPHS1, OTC, IVD*, and *PHOX2B*) were noted in more than four patients, accounting for 48.9% (64/131) of the identified P/LP variants. Forty-six diagnosed patients with uncorrected NMA died or gave up. In conclusion, 37% of neonates with metabolic acidosis had genetic disorders. Next-generation sequencing should be considered when investigating the etiology of NMA in NICUs. Based on early molecular diagnoses, valuable treatment options can be provided for some genetic diseases to achieve better outcomes.

## Introduction

Neonatal metabolic acidosis (NMA) is the accumulation of non-carbonic acid equivalents, which arises from excessive production or inadequate excretion of hydrogen ions or from an increased loss of bicarbonate (1). Neonatal metabolic acidosis is associated with poor clinical outcomes (2). The clinical features of NMA are atypical and are related to varying degrees of primary disease. The causes of NMA are complicated and varied, including birth asphyxia, cold stress, hypovolemia, sepsis, congenital heart disease, renal disease, and inborn errors of metabolism (1). Evaluation of detailed history, physical examination, and basic laboratory tests were insufficient to determine the cause. Administration of a fluid bolus or sodium bicarbonate is the initial management for correcting metabolic acidosis (3). These treatments do not deal with the cause but only correct the pH (3–6). Although interventions have been established in clinical practice, the impact on long-term neurological morbidity remains uncertain. With the increased awareness of the association between genotype and diseases, it has been found that infants with metabolic acidosis admitted to neonatal intensive care units (NICUs) are more likely to have genetic disorders. Thus, early genetic diagnosis is essential for neonates with NMA.

In recent years, next-generation sequencing (NGS) technology has been increasingly applied in clinical practice as a genetic diagnostic tool. Next-generation sequencing, especially exome sequencing and whole-genome sequencing, has shown advantages in facilitating an accurate diagnosis that is difficult to confirm using clinical or laboratory criteria for patients with disorders in the NICU (7, 8). Recent studies have reported enabling rapid sequencing at reduced costs and better performance in critically ill neonates (9, 10).

In this study, we analyzed the clinical medical records and genetic testing results of patients with NMA and summarized the clinical features, genetic diagnoses, and their effect on clinical intervention. Our study aims to provide a better understanding of the genetic causes of NMA that translate to more accurate management and better prognosis.

## Materials and Methods

### Study Design and Participants

This was a retrospective cohort study conducted at the Clinical Genetics Laboratory of the Children's Hospital of Fudan University. Patients in NICUs were recruited retrospectively between January 1, 2016, and December 31, 2019. The inclusion criteria were as follows: (1) diagnosis of NMA based on arterial blood gas, pH <7.3, BE < −10 mmol/L, and PaCO_2_ 35–45 mmHg; and (2) an order for NGS. Notably, patients with NMA who did not undergo NGS were excluded. In addition, patients were excluded if they were genetically diagnosed before enrollment. The clinical features of each patient were ascertained comprehensively by a physician in addition to a thorough review of their medical records. The clinical data included sex, major clinical features, and outcomes of metabolic acidosis. Pre-test counseling was performed by physicians. This study was approved by the Medical Ethics Committee of the Children's Hospital of Fudan University (2015-130). Informed consent was obtained from the patients' parents or guardians.

### Next-Generation Sequencing

Peripheral blood was collected, and genomic DNA was extracted using the TIANGEN DNA Blood Mini Kit according to the manufacturer's protocol. Sequences were generated using the Agilent ClearSeq Inherited Disease panel kit (including 2,742 genes) for clinical exome sequencing or the Agilent SureSelect XT Human All Exon V5 kit for exome sequencing. Next-generation sequencing was performed using the Illumina HiSeq X10 platform. The average on-target sequencing depth was × 200 for the clinical exome sequencing and × 120 for exome sequencing. Sequencing reads were mapped to the reference human genome (UCSC hg19) using the Burrows–Wheeler Aligner. A phenotype-scoring algorithm named PhenoPro was used for the variant filtering process (11). The detected variants were confirmed using PCR, and PCR-amplified DNA products were subjected to direct automated sequencing (3500XL Genetic Analyzer, Applied Biosystems) according to the manufacturer's specifications. *De novo* variants were detected by parental confirmation using Sanger sequencing. The pathogenicity of the variant was defined based on the American College of Medical Genetics and Genomics criteria (12). Detailed methods can be found in our previous studies (13, 14).

### Statistical Analysis

Frequency count and proportions were used for categorical data. Data were analyzed using Pearson's χ^2^ independence test. Statistical significance was set at a *p-*value < 0.05. All statistical analyses were conducted using IBM SPSS version 20 (IBM Corp., Armonk, NY, USA).

## Results

### Demographics of Clinical Features

**Figure 1:**
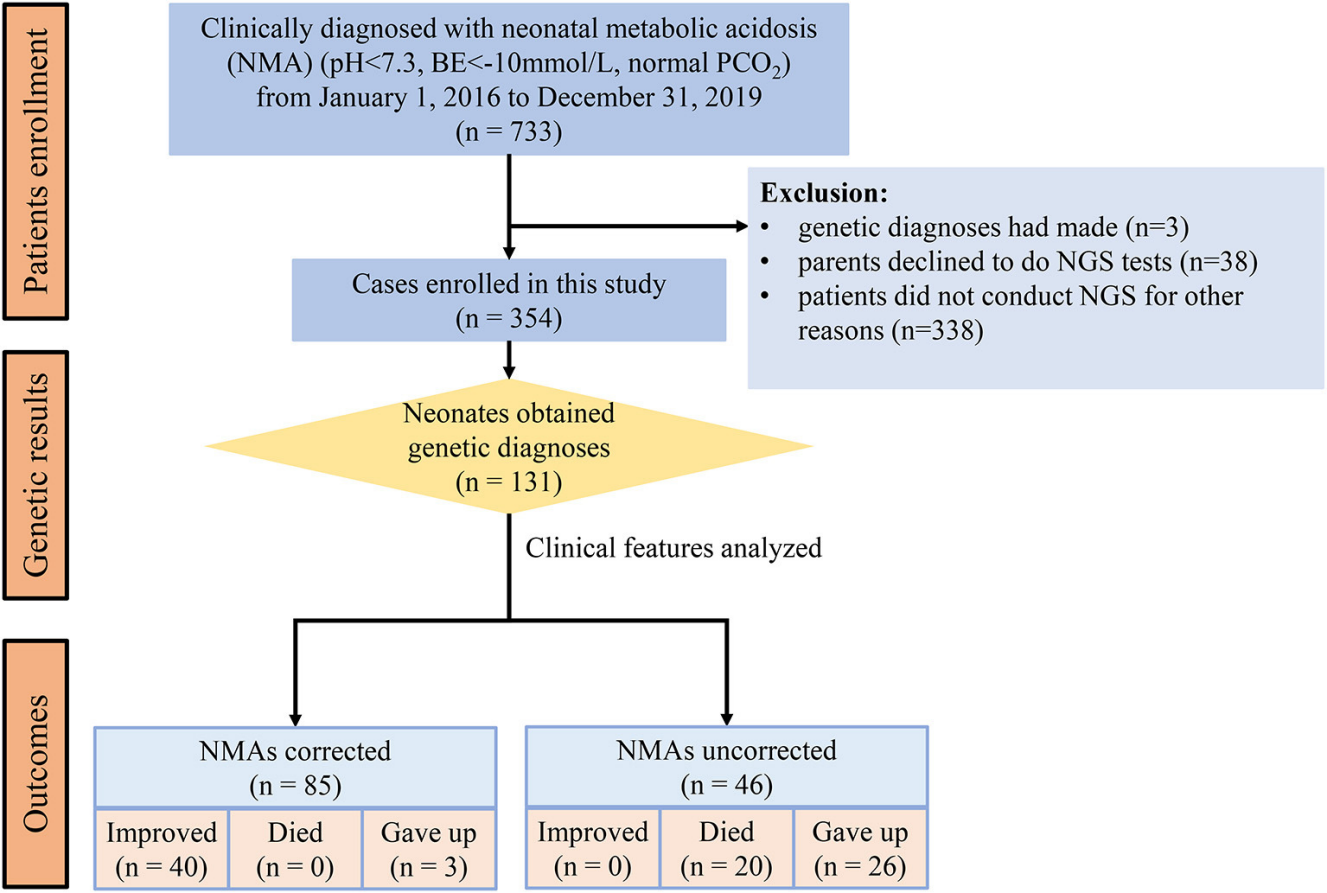
Flowchart of diagnosed patients by NGS.

From January 1, 2016, to December 31, 2019, 733 patients were clinically diagnosed with metabolic acidosis in NICUs. Three cases had genetic diagnoses, 38 neonates did not undergo NGS because their parents declined, and 338 patients did not undergo NGS for other reasons. Finally, 354 (48.3%) neonates with NGS were enrolled and classified as diagnosed and undiagnosed according to the genetic findings of our analysis (Figure 1). Of the enrolled patients, 186 (52.5%) were males and 168 (47.5%) were females. The major clinical manifestations (one patient may have more than one clinical feature) were neuromuscular dysfunction (49.2%), cardiopulmonary dysfunction (37.3%), hepatorenal disorder (17.2%), infection (13.6%), and malformation (12.1%) (Table 1).

**Table 1:** Demographic features and phenotypes of NMA patients tested with NGS.

**Characteristics**	**Total**	**Diagnosed**	**Undiagnosed**	***p-*value**
	***N* (%)**	***N* (%)**	***N* (%)**	
	**(Total = 354)**	**(Total = 131)**	**(Total = 223)**	
Sex				0.043
Male	186 (52.5)	78 (59.5)	108 (48.4)	
Female	168 (47.5)	53 (40.5)	115 (51.6)	
Major clinical features by systems^*^				
Neuromuscular dysfunction	174 (49.2)	56 (42.7)	118 (52.9)	0.065
Cardiorespiratory dysfunction	132 (37.3)	45 (34.3)	87 (39.0)	0.381
Hepatorenal disorder	61 (17.2)	22 (16.8)	39 (17.5)	0.867
Infection	48 (13.6)	20 (15.3)	28 (12.6)	0.472
Malformation	43 (12.1)	25 (19.1)	18 (8.1)	0.002
Outcomes of metabolic acidosis				0.63
Been corrected	224 (63.3)	85 (64.9)	139 (62.3)	
Uncorrected	130 (36.7)	46 (35.1)	84 (37.7)	
Overall outcomes				
Improved	149 (42.1)	40 (30.5)	109 (48.9)	0.001
Gave up medical support	55 (15.5)	29 (22.1)	26 (11.7)	0.009
Died with diseases	52 (14.7)	20 (15.3)	32 (14.3)	0.814

* *One patient may have more than one clinical feature. p-values are obtained from a χ^2^-test*.

### Genetic Diagnosis

Of these 354 neonates, 131 (37%) received genetic diagnoses through NGS, while the remaining did not (*n* = 223, 63%). The malformation rate was higher in the diagnosed group (9.1%, *p* = 0.002) than in the undiagnosed group (8.0%). However, there were no other significantly different major clinical features between the diagnosed and undiagnosed groups (Table 1).

In 131 patients with genetic diagnoses, 215 variants spanning 57 genes were classified as pathogenic (P) or likely pathogenic (LP). These patients were diagnosed with a monogenetic disorder, with 95 autosomal recessive cases, which included 80 compound heterozygous and 15 homozygous cases, 22 autosomal dominants with eight cases identified as *de novo*, 12 X-link recessive cases with hemizygote, and two X-link dominants with heterozygous cases.

**Figure 2:**
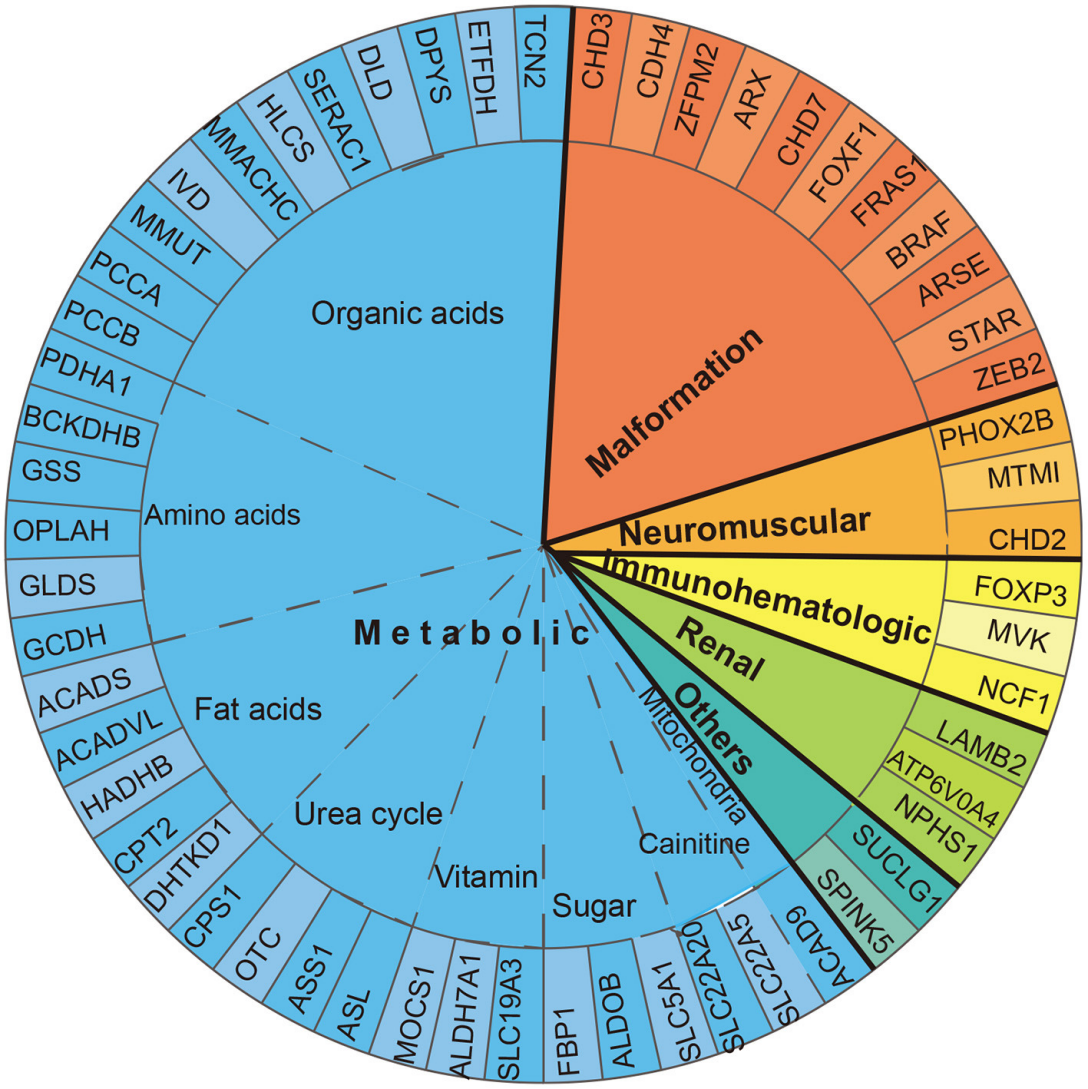
Diagnosed gene distribution according to the disease. Fifty-seven genes are displayed by classification. Thirty-five genes related to metabolic disorder were classified into eight subgroups: organic acid, amino acid, fat acid, urea cycle, sugar, vitamin, carnitine, and mitochondria disorder.

**Figure 3:**
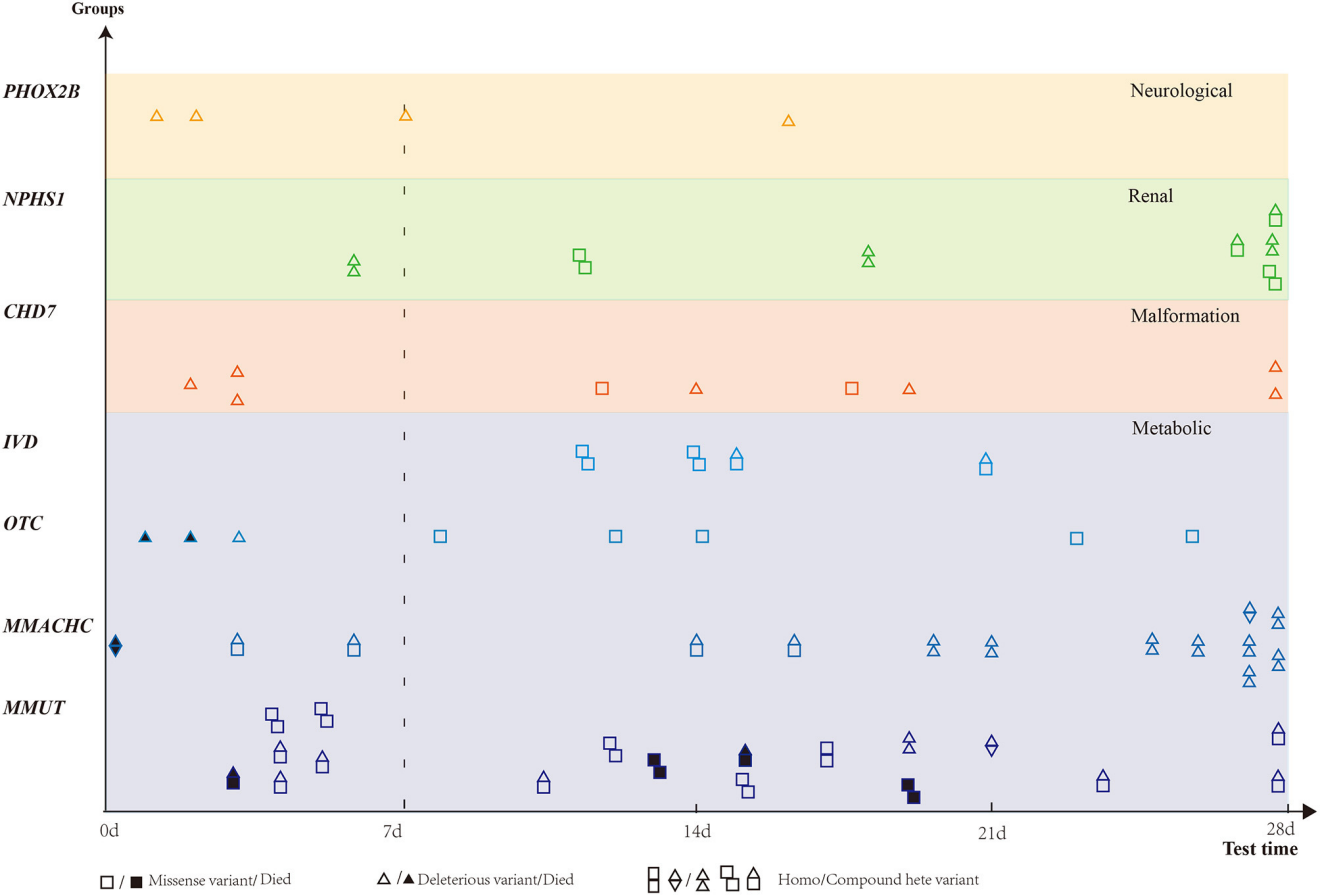
Distribution of genes detected in more than four patients. Six genes were classified into four groups based on diseases. X-axis presents blood gas test time. Patients with missense variant are denoted by square, and deleterious variants are denoted by a triangle. Death is denoted by a solid black square or triangle.

According to the 57 phenotype–genotype-related diagnoses, diseases were classified into six categories: metabolic, renal, neuromuscular, and immune-hematological disorders; malformation; and others. Metabolic disorder was the most common disease found in 89 (67.9%) patients, followed by malformation in 22 (16.8%) patients. Renal, neuromuscular, and immune-hematological disorders were detected in nine (6.9%), six (4.6%), and three (2.3%) patients, respectively. Two other genes were detected in two patients (Figure 2). Seven genes with P/LP variants identified in more than four patients were *MMUT, MMACHC, CHD7, NPHS1, OTC, IVD*, and *PHOX2B*, accounting for 48.9% (64/131) of the patients in our cohort (Figure 3).

Of the metabolic disorders, 35 genes were classified into eight subgroups: amino acids, organic acids, sugar, fatty acids, carnitine, urea cycle, vitamin, and mitochondria disorders. Eleven genes were responsible for organic acid disorders, covering 53.9% (48/89) of the metabolic disorder cases. Four genes (*OTC, ASL, CPS1*, and *ASS1*) were responsible for urea cycle disorders, accounting for 14.6% (13/89). Fatty acid, amino acid, and carnitine disorders were detected in eight (9%), eight (9%), and five (5.6%) patients, respectively. Sugar and vitamin disorders were found in three (3.3%) patients. Mitochondria disorders were found in a patient (1.1%) (Figure 2).

### Clinical Intervention and Outcomes

Metabolic acidosis was clinically corrected in 224 (63.3%) patients. However, metabolic acidosis was not corrected in 130 (36.7%) patients, including 46 diagnosed and 84 undiagnosed neonates. In the improved outcome cases, the rate of improvement was higher in the undiagnosed group (48.9%) than in the diagnosed group (30.5%) (*p* < 0.01). In contrast, the rate of patients who gave up medical support was higher in the diagnosed group (22.1 vs. 11.6%, *p* < 0.01) (Table 1). In the 131 diagnosed cases, 46 neonates with uncorrected NMAs all died or gave up. However, only three cases gave up, and no case died in the 85 neonates with corrected NMAs (Figure 1).

Of the 52 deaths in this study, 38.5% (20/52) received a genetic diagnosis (Table 1). Of these, 5 (25%) were diagnosed postmortem because their symptoms developed and progressed rapidly, resulting in mortality 24 h after admission. Through genetic diagnosis, accurate diagnoses can explain the deaths. *OTC* was the cause of death due to hyperammonemia in case NMA011 and encephalopathy and sepsis in case NMA047. Case NMA069, who carried two missense variants in *ACADVL*, died from cardiopulmonary arrest. Case NMA002 with encephalopathy and multiple organ failure was identified as a missense variant of GSS.

In addition, 40 patients with genetic diagnoses may receive precise treatment options. Similar to case NMA074, a 28-day-old male patient presented with poor feeding and lethargy. He was diagnosed with distal renal tubular acidosis after genetic identification with compound heterozygous variants in the *ATP6V0A4* gene. If the baby was not treated accurately and was followed up regularly, he would have developed severe complications, which would affect growth and development. Case NMA036, who was a 27-day-old male infant with lethargy and poor weight gain, was admitted to NICU. Blood tandem mass spectrometry showed increased tyrosine level, and the rest of the routine tests were highly suspicious for infection. Finally, methylmalonic acidemia (MMA) and homocystinuria, cblC type, were diagnosed based on genetic findings. The infant had improved upon targeted management. NMA003 was an 8-day-old baby boy who presented with vomiting, hypoglycemia, and seizures. A high level of tyrosine was found in blood mass spectrometry, and tyrosine metabolism disorder was more likely to be diagnosed. However, a hemizygote variation (G71E) was detected in the *OTC* gene by NGS and was classified as LP. The baby was diagnosed with ornithine transcarbamylase deficiency (OTCD, MIM 311250) based on genetic findings. The patient survived on a new effective treatment option. Case NMA111, who was a 20-day-old female infant with hepatic dysfunction and suspected sepsis, had compound heterozygous variants in the gene *ALDOB*. The patient was diagnosed with hereditary fructose intolerance (HFI, MIM 229600). The disease generally develops after feeding in the neonatal period. Severity is related to the amount and duration of fructose consumption. The baby was treated immediately with a fructose intake-controlled diet, which can prevent life-threatening complications and avoid invasive procedures such as liver biopsy. The baby can grow into adulthood on a well-controlled diet.

## Discussion

In our study, 131 of 354 (37%) neonates with metabolic acidosis received a genetic diagnosis. In total, 57 genes were responsible for a wide range of diseases, from seemingly healthy infants to critically ill patients. Different degrees of primary disease lead to varied and atypical clinical presentations. Complex etiologies and non-specific clinical signs made diagnosis more difficult.

The leading cause of NMA was metabolic disorders, which involved 35 genes, covering 68% of genetically diagnosed patients. Only 38 (38/89, 42.9%) patients had clinical diagnoses before receiving genetic diagnoses. Organic acid disorder is the major cause of metabolic acidosis. The top two genes, *MMUT* and *MMACHC*, resulted in MMA that was related to organic acid disorder. A total of 24 (24/32, 75%) MMA patients were diagnosed with MMA using mass spectrometric tests before NGS. Next-generation sequencing can help confirm diagnoses and determine the subtype of organic acid disorder. Phenotype–genotype correlations may predict disease severity depending on mRNA stability and protein residual function (15). Variations in *MMUT* were identified as MMUT(0) phenotypes, indicating no detectable enzymatic activity (16). Variations in *MMACHC* have been detected as MMA and homocystinuria, cblC type (15). Patients who are compound heterozygotes for a missense allele appear to have a milder phenotype (15). Variation analysis was associated with responses to vitamin B12. Generally, the cblC type is almost entirely vitamin B12 responsive, the MMUT(0) type is vitamin B12 unresponsive, and other types are partly responsive to vitamin B12 (17). *IVD* variations were found in four patients; three of them were diagnosed with isovaleric acidemia by mass spectrometric tests before NGS. Genetic findings confirmed the diagnoses and treatment. *OTC* variation results in OTCD, which accounts for approximately half of the urea cycle defects (18). As our data presented, *OTC* variations were detected in eight patients. Three of these patients were diagnosed with urea cycle defects by mass spectrometric tests before NGS. Genetic findings may help physicians understand the progress of OTCD. Six patients with missense variants that can reduce OTC enzymatic activity or stability survived OTCD (18). Patient NMA047 died 24 h after admission. He was found to carry a splicing variant, affecting mRNA processing and decreasing OTC enzyme levels (18).

Interestingly, in 42 diagnosed patients (42/131, 32%), NMA was caused by malformation and renal, neuromuscular, and immune-hematological disorders, and not by metabolic disorders. P/LP variations in *NPHS1*, which encodes nephrin, were detected in seven patients with massive proteinuria, edema, infection, and poor feeding or respiratory distress. NPHS1 is the main underlying cause of congenital nephritic syndrome, which is associated with high morbidity and mortality (19). Genetic diagnosis provides precise information on treatment and benefits patient outcomes. Pathogenic variants of *CHD7* were detected in nine neonates with infection, feeding difficulty, respiratory distress, and one or more malformations, which led to severe metabolic acidosis. These patients should be treated as if they are diagnosed with CHARGE syndrome (20). However, physicians will not give the diagnosis of CHARGE syndrome without genetic basis because criteria that focus on typical clinical phenotypes may exclude patients with a mild phenotype in the neonatal period. It has been proposed that pathogenic *CHD7* variant status is now a major criterion in CHARGE syndrome diagnoses (21). In four patients with recurrent apnea, infection, hypoxemia, and pathogenic variants, *PHOX2B* was identified. Genetic tests help physicians and families find the cause of diseases and help predict progress thereafter (22). Our study showed that mass spectrometric tests were insufficient for investigating the etiology of NMA, as opposed to NGS.

Based on early molecular diagnoses, valuable treatment options can be provided for some genetic diseases, and patients can survive or achieve better outcomes. For case NMA074, genetic diagnosis (distal renal tubular acidosis) was performed before the appearance of severe complications. With earlier adequate metabolic control, the infant can experience better growth and kidney function (23). Case NMA003, an 8-day-old male infant with abnormal mass spectrometric tests, was highly suspected to have metabolic disorder. OTCD was confirmed by genetic testing and appropriately treated. Case NMA036, a 27-day-old infant, underwent many blood tests and invasive procedures. The infant did not improve on anti-infection treatment but did improve with targeted treatment of MMA, which was diagnosed by NGS. For case NMA111, the female infant only developed clinical symptoms when she was exposed to fructose as a monosaccharide, sucrose, or sorbitol (24). The patient recovered after a fructose-controlled diet, and liver biopsy was no longer necessary. These are the most desirable results for physicians and families and help push research into new therapies.

Although there was no difference between diagnosed and undiagnosed patients, if we compare the outcomes of NMA, the genetic test can effectively identify the etiology of the disease, and these precise diagnoses enable physicians and families to understand the cause of disease and the unavoidable poor clinical outcomes (25). Genetic counseling for families provided detailed progress, helped parents identify family carriers, and facilitated better planning of reproductive choices based on the risk of familial recurrence (26). The families in our study may benefit from previous genetic diagnoses and genetic counseling.

Based on our analysis, early genetic diagnosis is essential for neonates with NMA. We proposed that NGS be performed as early as possible when patients suffer from uncorrected metabolic acidosis and combine with one of the following items: (1) abnormal blood mass spectrometry or other abnormal biochemical markers such as blood ammonia or blood lactic acid; and (2) NMA neonates accompanied with malformation. For other NMA neonates, we suggest that NGS tests such as clinical exome sequencing should be ordered when physicians think that the NMA is a genetic factor involved.

This study had two limitations that should be addressed. First, although we designed to test all NMA patients in the NICU, we had 338 cases that did not undergo NGS for different reasons. Second, as a multicenter study, follow-up information was limited, and the outcomes of some patients were missed.

## Conclusion

In our study, we found that metabolic acidosis in approximately 37% of neonates in NICU was caused by genetic disorders. Next-generation sequencing should be considered when investigating the etiology of NMA. Based on early molecular diagnoses, valuable treatment options can be provided for some genetic diseases to achieve better outcomes.

## Ethics Statement

The studies involving human participants were reviewed and approved by Medical Ethics Committee of Children's Hospital of Fudan University (2015-130). Written informed consent to participate in this study was provided by the participants' legal guardian/next of kin. Written informed consent was obtained from the minor(s)' legal guardian/next of kin for the publication of any potentially identifiable images or data included in this article.

## Author Contributions

HW, DZ, and GL designed and supervised the overall study. FX, YLu, XD, GC, LW, WL, LY, QN, XP, and YW conducted analysis on the aggregated large cohort data. LL, YLi, WT, LC, WK, YC, BW, and WZ collected, supervised, and reviewed the clinical data. HM and ZT wrote the original manuscript draft. HW critically reviewed and revised the manuscript for important intellectual content. All authors approved the final manuscript as submitted and agreed to be accountable for all aspects of the work.

## Conflict of Interest

The authors declare that the research was conducted in the absence of any commercial or financial relationships that could be construed as a potential conflict of interest.

## Publisher's Note

All claims expressed in this article are solely those of the authors and do not necessarily represent those of their affiliated organizations, or those of the publisher, the editors and the reviewers. Any product that may be evaluated in this article, or claim that may be made by its manufacturer, is not guaranteed or endorsed by the publisher.

## References

[1] Walter JH. Metabolic acidosis in newborn infants. Arch Dis Child. (1992) 67(7 Spec No):767–9. 10.1136/adc.67.7_spec_no.767 1519972 PMC1590423

[2] Kraut JA Madias NE. Treatment of acute metabolic acidosis: a pathophysiologic approach. Nat Rev Nephrol. (2012) 8:589–601. 10.1038/nrneph.2012.186 22945490

[3] Lawn CJ Weir FJ McGuire W. Base administration or fluid bolus for preventing morbidity and mortality in preterm infants with metabolic acidosis. Cochrane Database Syst Rev. (2005) (2):CD003215. 10.1002/14651858.CD003215.pub2 15846651 PMC8711593

[4] Aschner JL Poland RL. Sodium bicarbonate: basically useless therapy. Pediatrics. (2008) 122:831–5. 10.1542/peds.2007-2400 18829808

[5] Rochwalsky U Seitz C Heinzmann T Poeschl J Koch L. [Correction of acidosis in neonatal intensive-care medicine: a national survey]. Klin Padiatr. (2015) 227:219–24. 10.1055/s-0034-1396866 25811742

[6] Collins A Sahni R. Uses and misuses of sodium bicarbonate in the neonatal intensive care unit. Semin Fetal Neonat Med. (2017) 22:336–41. 10.1016/j.siny.2017.07.010 28801177

[7] Meng L Pammi M Saronwala A Magoulas P Ghazi AR Vetrini F. Use of exome sequencing for infants in intensive care units: ascertainment of severe single-gene disorders and effect on medical management. JAMA Pediatr. (2017) 171:e173438. 10.1001/jamapediatrics.2017.3438 28973083 PMC6359927

[8] Smith HS Swint JM Lalani SR Yamal JM de Oliveira Otto MC Castellanos S. Clinical application of genome and exome sequencing as a diagnostic tool for pediatric patients: a scoping review of the literature. Genet Med. (2019) 21:3–16. 10.1038/s41436-018-0024-6 29760485

[9] Elliott AM du Souich C Lehman A Guella I Evans DM Candido T. RAPIDOMICS: rapid genome-wide sequencing in a neonatal intensive care unit-successes and challenges. Eur J Pediatr. (2019) 178:1207–18. 10.1007/s00431-019-03399-4 31172278

[10] Wang H Lu Y Dong X Lu G Cheng G Qian Y. Optimized trio genome sequencing (OTGS) as a first-tier genetic test in critically ill infants: practice in China. Hum Genet. (2020) 139:473–82. 10.1007/s00439-019-02103-8 31965297

[11] Li Z Zhang F Wang Y Qiu Y Wu Y Lu Y. PhenoPro: a novel toolkit for assisting in the diagnosis of Mendelian disease. Bioinformatics. (2019) 35:3559–66. 10.1093/bioinformatics/btz100 30843052

[12] Richards S Aziz N Bale S Bick D Das S Gastier-Foster J. Standards and guidelines for the interpretation of sequence variants: a joint consensus recommendation of the American College of Medical Genetics and Genomics and the Association for Molecular Pathology. Genet Med. (2015) 17:405–24. 10.1038/gim.2015.30 25741868 PMC4544753

[13] Yang L Kong Y Dong X Hu L Lin Y Chen X. Clinical and genetic spectrum of a large cohort of children with epilepsy in China. Genet Med. (2019) 21:564–71. 10.1038/s41436-018-0091-8 29930392 PMC6681813

[14] Wang H Xiao F Dong X Lu Y Cheng G Wang L. Diagnostic and clinical utility of next-generation sequencing in children born with multiple congenital anomalies in the China neonatal genomes project. Hum Mutat. (2021) 42:434–44. 10.1002/humu.24170 33502061

[15] Carrillo-Carrasco N Chandler RJ Venditti CP. Combined methylmalonic acidemia and homocystinuria, cblC type. I. Clinical presentations, diagnosis and management. J Inherit Metab Dis. (2012) 35:91–102. 10.1007/s10545-011-9364-y 21748409 PMC4219318

[16] Almási T Guey LT Lukacs C Csetneki K Vokó Z Zelei T. Systematic literature review and meta-analysis on the epidemiology of methylmalonic acidemia (MMA) with a focus on MMA caused by methylmalonyl-CoA mutase (mut) deficiency. Orphan J Rare Dis. (2019) 14:84. 10.1186/s13023-019-1063-z 31023387 PMC6485056

[17] Zhou X Cui Y Han J. Methylmalonic acidemia: current status and research priorities. Intract Rare Dis Res. (2018) 7:73–8. 10.5582/irdr.2018.01026 29862147 PMC5982627

[18] Caldovic L Abdikarim I Narain S Tuchman M Morizono H. Genotype-phenotype correlations in ornithine transcarbamylase deficiency: a mutation update. J Genet Genomics. (2015) 42:181–94. 10.1016/j.jgg.2015.04.003 26059767 PMC4565140

[19] Sharief SN Hefni NA Alzahrani WA Nazer II Bayazeed MA Alhasan KA. Genetics of congenital and infantile nephrotic syndrome. World J Pediatr. (2019) 15:198–203. 10.1007/s12519-018-00224-0 30721404

[20] Hsu P Ma A Wilson M Williams G Curotta J Munns CF. CHARGE syndrome: a review. J Paediatr Child Health. (2014) 50:504–11. 10.1111/jpc.12497 24548020

[21] Hale CL Niederriter AN Green GE Martin DM. Atypical phenotypes associated with pathogenic CHD7 variants and a proposal for broadening CHARGE syndrome clinical diagnostic criteria. Amer J Med Genet A. (2016) 170A:344–54. 10.1002/ajmg.a.37435 26590800 PMC5102387

[22] Bishara J Keens TG Perez IA. The genetics of congenital central hypoventilation syndrome: clinical implications. Appl Clin Genet. (2018) 11:135–44. 10.2147/TACG.S140629 30532577 PMC6241683

[23] Lopez-Garcia SC Emma F Walsh SB Fila M Hooman N Zaniew M. Treatment and long-term outcome in primary distal renal tubular acidosis. Nephrol Dial Transplant. (2019) 34:981–91. 10.1093/ndt/gfy409 30773598

[24] Tran C. Inborn errors of fructose metabolism. What can we learn from them? Nutrients. (2017). 9:356. 10.3390/nu9040356 28368361 PMC5409695

[25] Bowdin SC. The clinical utility of next-generation sequencing in the neonatal intensive care unit. CMAJ. (2016) 188:786–7. 10.1503/cmaj.160490 27241783 PMC4978569

[26] Borghesi A Mencarelli MA Memo L Ferrero GB Bartuli A Genuardi M. Intersociety policy statement on the use of whole-exome sequencing in the critically ill newborn infant. Ital J Pediatr. (2017) 43:100. 10.1186/s13052-017-0418-0 29100554 PMC5670717

